# Transport of pregnant women and obstetric emergencies in India: an analysis of the ‘108’ ambulance service system data

**DOI:** 10.1186/s12884-016-1113-7

**Published:** 2016-10-21

**Authors:** Samiksha Singh, Pat Doyle, Oona M. R. Campbell, G. V. R. Rao, G. V. S. Murthy

**Affiliations:** 1 Indian Institute of Public Health-Hyderabad, Public Health Foundation of India, Hyderabad, Telangana India; 2 Department of Non-communicable Disease Epidemiology, Faculty of Epidemiology and Population Health, London School of Hygiene and Tropical Medicine, London, UK; 3 Department of Infectious Disease Epidemiology, Faculty of Epidemiology and Population Health, London School of Hygiene and Tropical Medicine, London, UK; 4 GVK-EMRI, Hyderabad, Telangana India; 5 Department of Clinical Research, Faculty of ITD, London School of Hygiene and Tropical Medicine, London, UK

**Keywords:** Ambulance, Obstetric emergency, Obstetric complication, Pregnant women, Patient transport, Access, Maternity Services, Travel time

## Abstract

**Background:**

The transport of pregnant women to an appropriate health facility plays a pivotal role in preventing maternal deaths. In India, state-run call-centre based ambulance systems (‘108’ and ‘102’), along with district-level Janani Express and local community-based innovations, provide transport services for pregnant women. We studied the role of ‘108’ ambulance services in transporting pregnant women routinely and obstetric emergencies in India.

**Methods:**

This study was an analysis of ‘108’ ambulance call-centre data from six states for the year 2013–14. We estimated the number of expected pregnancies and obstetric complications for each state and calculated the proportions of these transported using ‘108’. The characteristics of the pregnant women transported, their obstetric complications, and the distance and travel-time for journeys made, are described for each state.

**Results:**

The estimated proportion of pregnant women transported by ‘108’ ambulance services ranged from 9.0 % in Chhattisgarh to 20.5 % in Himachal Pradesh. The ‘108’ service transported an estimated 12.7 % of obstetric emergencies in Himachal Pradesh, 7.2 % in Gujarat and less than 3.5 % in other states. Women who used the service were more likely to be from rural backgrounds and from lower socio-economic strata of the population. Across states, the ambulance journeys traversed less than 10–11 km to reach 50 % of obstetric emergencies and less than 10–21 km to reach hospitals from the pick-up site. The overall time from the call to reaching the hospital was less than 2 h for 89 % to 98 % of obstetric emergencies in 5 states, although this percentage was 61 % in Himachal Pradesh. Inter-facility transfers ranged between 2.4 % –11.3 % of all ‘108’ transports.

**Conclusion:**

A small proportion of pregnant women and obstetric emergencies made use of ‘108’ services. Community-based studies are required to study knowledge and preferences, and to assess the potential for increasing or rationalising the use of ‘108’ services.

## Background

Most maternal deaths could be prevented if women are assisted by skilled attendants at birth and those experiencing complications could reach quality emergency obstetric care (EmOC) in a timely manner [1]. It is estimated that at least 15 % of all pregnancies will encounter complications and 7 % will be serious enough to require referral to a higher level of care [1]. In many countries with high maternal mortality the lack of transport, poor communication, high cost and geographical barriers can lead to fatal delays in reaching life-saving care [2]. Delays in reaching basic delivery care and EmOC also contribute to maternal morbidity in the form of medical complications, obstetric fistula, disability, and depression, as well as perinatal mortality [2]. Making transport freely available to pregnant women is thus a key intervention to reduce such delays [3, 4]. Many such interventions have proven successful and these include adequate birth planning, availability and low costs for transport, along with effective communication systems [5].

In 2013, India had an estimated institutional delivery rate of 82.7 % [6], a maternal mortality ratio of 167 per 100,000 live births [7] and early neo-natal mortality rate of 28 per 1000 live births [7]. Although India has shown substantial improvement in these indicators in the last decade [8–10], but it has failed in achieving MDG-4 and MDG-5 targets for the reduction in child and maternal mortality by 2015 [11, 12]. Failure to meet these targets is explained by lack of access to appropriate specialist care, poor quality of care and poor systems of transport for high-risk pregnancies or obstetric complications to EmOC- contributing to the failure of timely access [13]. Studies in India reveal that about one-third to one-half of reported maternal deaths occurred at home or on the way to care [14–16].

Since 2002, the government has implemented many schemes and interventions to improve basic transport and ambulances for pregnant women under the Reproductive and Child Health-2 Program and the National Rural Health Mission [17]. These include the Janani Suraksha Yojana- a cash transfer scheme that covers some travel costs; regional voucher schemes; state run call centre based ambulance systems (‘108’ and ‘102’); and decentralised district level public private partnerships such as Janani Express and local community-based innovations to provide basic transport services [18, 19]. In addition, the Janani Shishu Suraksha Karyakaram scheme works mainly through the ‘102’ ambulance system to provide reverse transport from a facility to home for the poor [20].

The ‘108’ call centre based ambulance system is a free of cost emergency response system, known to be one of the largest public private partnership (PPP) initiatives across India functioning in 20 states and two union territories. Although it is designed primarily to attend to patients who are critically ill and, victims of trauma and accidents, state governments consider ‘108’ ambulance services to be the mainstay of transport for pregnant women in both normal labour and emergency [21]. The service is provided in partnership with three private institutes: the GVK-Emergency Medicine Research Institute (GVK-EMRI) which serves in 15 states and two union territories; the Ziqitza Health Care Limited in four states; and the Bharat Vikas Group Limited in one state [21].

The role of the ‘108’ ambulance service in reducing maternal mortality and severe morbidity is currently unknown. The aim of this research is to contribute to knowledge about the use of the ‘108’ ambulance service for pregnant women in India. The objectives of the research were to (i) describe the characteristics of pregnant women who requested ‘108’ assistance, (ii) to estimate the proportion of all pregnant women and obstetric emergencies who made use of the ‘108’ service, (iii) to describe the characteristics of the women transported, their obstetric complications, and the journeys made, for each state.

## Methods

### Context

GVK-EMRI, the first and largest service provider of ‘108’ was chosen for this study. It started operations in August 2005 in Andhra Pradesh and spread to other states. The proportion of all ‘108’ transports that were pregnancy related increased from 2 % in 2006 to 21 % in 2009 in Andhra Pradesh [22]. Across India also, this rose dramatically from 2 % in 2005–06 to 41.2 % in 2014–15 [23].

According to the operational guidelines of the ‘108’ ambulance service, there should be about one ambulance per 100,000 population. The ambulances should be well equipped and accompanied by a trained Emergency Medicine Technician (EMT) who could provide pre-hospital care before transfer. Pregnant women can be provided with intravenous fluids and oxygen if required, and magnesium-sulphate and oxytocin after consulting the call-centre based medical officer. In case of imminent childbirth, the EMT can assist the delivery at home or en-route, and transfer the mother and child to the nearest health facility [24]. In the ‘108’ system every pregnancy is a priority and there is no triage for emergencies in pregnancy or the postpartum period. Ambulances are dispatched to only about 8 % of general health care calls (87 % of the remaining calls are irrelevant or mischief calls) compared to nearly 95 % or more for pregnancy related calls [25].

As a policy ‘108’ ambulances transport clients to the nearest appropriate public health institution. If there is none close to the pick-up site then they transport the client to the nearest private hospital that is empanelled under the ‘108’ emergency response system. The patient’s choice is also considered while making the decision. The ‘108’ ambulance service aims to reach patients/sites within 20 min in urban, and within 40 min in rural, areas and reach the nearest health facility within 20 min following pick-up [26].

### Study design

This study is a cross-sectional analysis of 108 ambulance records from six states for one year. The numbers, proportions, and characteristics of pregnant women and obstetric emergencies transported by the ‘108’ ambulance service are described.

Obstetric emergency, for this study, is defined as any life-threatening medical complication related to pregnancy or a medical condition complicating pregnancy- during pregnancy, labour or child-birth, or within 42 days of termination of pregnancy. A pregnancy-related call is defined as any call from, or for a pregnant woman, in labour or in the post-partum period for antenatal care, abortion related care, labour pains, child-birth, post-birth care in the postpartum period, or any complication in these periods.

### Study population

Pregnant women who called ‘108’ between 1st April 2013 and 31st March 2014 in five states where GVK-EMRI had been fully functional for more than 3 years were included in this analysis. One state was selected randomly from North, South, Central, West and East of India. These were Himachal Pradesh, (undivided) Andhra Pradesh, Chhattisgarh, Gujarat, and Assam. Andhra Pradesh was subsequently officially divided into Telangana and (new) Andhra Pradesh in June 2014. During analysis, data were segregated and analysed separately for Telangana and (new) Andhra Pradesh and thus in the rest of the paper we describe six states.

### Obtaining data

GVK-EMRI emergency response centre records basic information about the client when he/she calls ‘108’ for an ambulance. Later, after examination by the EMT and once the case is transported, the EMT reports to the emergency response centre to provide more information on social-economic status, clinical condition, and treatment provided en-route, and details of journeys undertaken.

Official permission to use the data was obtained from GVK-EMRI and ethical approval for the study was obtained from Indian Institute of Public Health-Hyderabad and the London School of Hygiene and Tropical Medicine. Anonymised information on ‘108’ calls from 1st April 2013 to 31st March 2014 was obtained from the GVK-EMRI emergency response centre database. No personal identifiers were recorded. Data were linked with the incident/case id recorded by the call centre. Variables of interest for the secondary data analysis were: type of call; ambulance assigned or not; assigned ambulance used or not; type of emergency; age; social class; economic class; region; time of call; day of call; time taken by ambulance to reach the client; time taken to reach the health facility; distance travelled; inter-facility transfers; and mortality.

### Data management

Data were extracted from a central database onto Excel sheets, and analysis was done using STATA 13.0. Data were inspected before use to assess consistency, range, and missing data. Any gross issue related to the quality of records was noted. Where appropriate, variables were recoded. About 1 –5 % data were missing in most of the variables. However for social-class and economic status, 10 –30 % of the data was missing. There were a few data inconsistencies. Some possible wrong entries were noted in the time and distance variables but these constituted less than 0.5 % of all data. These records were excluded from analyses. The states included in the study also transported neonates in the post-partum period, and in Telangana and (new) Andhra Pradesh, mothers and newborns were also transported back from hospital to home. This information was extracted from 3 variables and text remarks within the dataset and these cases were excluded from analysis.

The ‘108’ annual reports also classify delivery by EMT at home or in the ambulance, suicide/ injury/ accidents and category ‘others’ as emergencies. Deliveries assisted by EMTs did not have information on whether these were normal delivery cases or had complications. EMTs were trained only to handle normal deliveries. We were not sure whether suicide/ injury/ accidents were classified as medical conditions due to pregnancy, or complicated by pregnancy or otherwise, as there was no further information on these in the EMT logs. Since we could not reliably classify these cases as obstetric emergences we excluded them from this analysis.

### Analysis

#### Characteristics of state populations and pregnant women who requested 108 assistance

Socio-demographic information for the populations in six states were collated from the Census, the Sample Registration System (SRS), District Level Household Survey (DLHS), and Annual Health Survey (AHS), using information as close to the study period dates as possible. Information on pregnancy related calls to ‘108’ were estimated from ‘108’call centre data (2013–2014) and presented by state. This information included whether or not the ambulance was assigned, or used. The characteristics of the callers were compared to the characteristics of the state populations.

#### Estimation of proportions of all pregnant women and obstetric emergencies who used the 108 service within states

For each state, the number of pregnancies expected in the study period was estimated as [population (rural) X crude birth rate (rural) X 1.1 × 1000] + [population (urban) X crude birth rate (urban) X 1.1 × 1000]. The population data were obtained from the 2011 census and the crude birth rates from the Sample Registration System 2013. The multiplier 1.1 was used to account for an estimated 10 % of the pregnancies which may have ended in abortions or intra-uterine deaths.

The number of obstetric emergencies expected in the study period was estimated as [estimated no. of pregnancies X 0.15], using 15 % as the expected overall prevalence of direct obstetric emergencies.

The numbers of pregnant women transported by ‘108’ as recorded in the call-centre database were compared with these estimated numbers for each state.

#### Characteristics of the women transported, their obstetric complications, and the journeys made, for each state

Socio-economic information on women who were transported using 108 ambulances was collated and presented by state. Clinical information on their obstetric emergencies, and the journeys taken by women experiencing obstetric emergencies, was also collated and presented according to state.

## Results

As background information, the characteristics of states with respect to population size, fertility and mortality were compiled (Table 1). Chhattisgarh and Assam are the poor performing states in maternal and child health. In Chhattisgarh, ‘108’ worked to full capacity for transporting pregnant women until September 2013 when ‘102’ ambulance service took over and the transports by ‘108’ reduced to 1/10th of the previous transfers, in October 2013 – March 2014.

**Table 1:** Demographic characteristics and use of ‘108’ ambulance in different states

	Telangana	Andhra Pradesh	Himachal Pradesh	Chhattisgarh	Gujarat	Assam
Demographic Characteristics						
Total Population^a^	35,193,978	49,386,799	6,856,509	25,540,196	60,383,628	31,169,272
Rural/tribal	61.3 %	70.4 %	90.0 %	76.8 %	57.4 %	85.9 %
Urban	38.7 %	29.6 %	10.0 %	23.2 %	42.6 %	14.1 %
Scheduled caste^a^	15.4 %	17.1 %	25.2 %	12.8 %	6.8 %	7.2 %
Scheduled tribe^a^	9.3 %	5.33 %	5.7 %	30.6 %	14.8 %	12.5 %
Crude birth rate per 1000 population^b^	17.5		16.2	24.5	21.1	22.5
Rural/tribal	17.9		16.7	26.0	22.5	23.7
Urban	16.6		11.0	18.0	18.7	15.6
Institutional delivery rate(2012–13)^c^	94.1 %	88.5 %	77.8 %	39.5 %	-awaited-	65.9 %
MMR(2011–13)^b^	92		-	244	112	300
NMR(2012–13)^b^	25		25	31	26	27
No. of Ambulances under ‘108’^d^	802		171	240	506	380
Pregnancy-related 108 Calls- April 2013 to March 2014						
Pregnancy related calls to ‘108’	122,619	172,076	25,016	65,243^e^	261,702	Not available
Area						
Rural/tribal	74.0 %	76.5 %	80.9 %	92.8 %	88.5 %	Not available
Urban	25.9 %	23.3 %	6.2 %	7.2 %	11.5 %	
Missing	0.2 %	0.2 %	12.9 %	0.0 %	0.0 %	
Use of ambulance						
Ambulance used	90.0 %	89.8 %	99.8 %	95.2 %	99.4 %	Not available
Ambulance not used	7.8 %	8.2 %	0.2 %	3.2 %	0.0 %	
Ambulance not assigned	2.2 %	2.0 %	0.0 %	1.6 %	0.6 %	

Source- ^a^Census 2011

^b^Sample Registration System 2013- Separate data for Telangana and Andhra Pradesh not available

^c^DLHS (2012–13) / AHS (2011–12)

^d^GVK-EMRI annual report for period April 2013- March 2014

^e^In Chhattisgarh ‘102’ ambulance service took over from October 2013 – March 2014

### Pregnancy related calls to ‘108’

A total of 621,640 pregnancy related calls were attended by the ‘108’ call centre in five states from April 2013 to March 2014. Data regarding these was not available from state of Assam. A higher proportion of calls were from rural than urban areas in all the states except Himachal Pradesh, even higher than expected from population proportions. For example, the rural population in Chattisgarh and Assam were 76.7 % and 57.4 % respectively (Table 1) while the proportion of ‘108’ service calls from rural areas in these two states were 92.8 % and 88.5 % respectively (Table 1).

Ambulances were assigned for more than 98 % of the pregnancy-related calls overall. Table 1 shows that the proportion of calls resulting in an ambulance not being assigned, and an ambulance not being used despite being assigned, was highest in Telangana and (new) Andhra Pradesh. The proportion of callers who were not assigned an ambulance did not vary between rural and urban populations, or between and non-IFT calls (data not shown). The proportion who did not use an ambulance (despite being assigned) was higher amongst urban (4.5 %) compared to rural (3.1 %) populations and in IFT (6.1 %) compared to non-IFT (3.9 %) callers.

### Estimated proportions of pregnant women, and obstetric emergencies, transported by ‘108’

Table 2 presents estimates of the proportion of pregnant women, and obstetric emergencies, transported using ‘108’. In total 757,697 pregnant women were transferred to hospitals using ‘108’ ambulances in the study year, which was 16.5 % of the estimated pregnancies for all the study states. The estimated proportion of pregnant women transported by ‘108’ ranged from 9.0 % in Chhattisgarh to 20.5 % in Himachal Pradesh.

**Table 2:** Estimated proportion of pregnant women transported to hospitals by ‘108’ April 2013 to March 2014

	Telangana	Andhra Pradesh	Himachal Pradesh	Chhattisgarh^c^	Gujarat	Assam
Estimated number of pregnancies in state^a^	673,508	950,696	121,638	678,217	1,387,021	773,480
-Transported by ‘108’	105,381 (15.7 %)	147,374 (15.5 %)	24,923 (20.5 %)	60,810 (9.0 %)	270,071 (19.5 %)	149,138 (19.3 %)
Estimated number of obstetric emergencies in state^b^	101,026	142,730	18,246	101,733	208,053	116,022
-Transported by ‘108’	3,570 (3.5 %)	4,837 (3.4 %)	2,316 (12.7 %)	2,660 (2.6 %)	15,065 (7.2 %)	4,040 (3.5 %)

^a^[population (rural) X crude birth rate (rural) X 1.1 × 1000] + [population (urban) X crude birth rate (urban) X 1.1 × 1000]

^b^Estimated no. of pregnancies X 0.15

^c^In Chhattisgarh ‘102’ ambulance service took over from October 2013 – March 2014

Overall, 4.7 % of expected obstetric emergencies (based on 15 % of all pregnancies in the population) were transported across the States. The highest estimated proportion of obstetric emergencies transported was in Himachal Pradesh (12.7 %) and Gujarat (7.2 %) (Table 2). For other states the estimated proportion of obstetric emergencies transported was 3.5 % or less.

### Characteristics of pregnant women transported by ‘108’

Table 3 describes the characteristics of pregnant women transported by the ‘108’ service. The majority were 20–35 years old. A higher proportion of users belonged to scheduled caste and scheduled tribes compared to the census population in all the states, except Chhattisgarh and Assam. Almost all the users in Telangana and Andhra Pradesh, 85 % in Assam, and about three fifths in Himachal Pradesh and Gujarat were classified as belonging to below the poverty line category. Between 2.4 % of pregnant women transported by ‘108’ ambulances in Gujarat, and 11.3 % in Himachal Pradesh, were referred and transported from a health institution. Less than 5 % deliveries happened at the pick-up site or in the ambulance en-route across the states. Between 2.7 % and 9.3 % of transported pregnant women had an obstetric emergency in the study states and the majority used ‘108’ ambulances for normal labour pains or other reasons.

**Table 3:** Characteristics of pregnant women transported to hospitals by ‘108’ April 2013 to March 2014

	Telangana *N* = 105,381	Andhra Pradesh *N* = 147,374	Himachal Pradesh *N* = 24,923	Chhattisgarh *N* = 60,810	Gujarat *N* = 270,071	Assam *N* = 149,138
Age, %						
< 20 years	3.6	4.9	5.5	5.8	1.7	8.1
20-35 years	95.6	94.2	91.2	92.7	79.7	87.6
> 35 years	0.5	0.6	1.5	1.0	1.0	0.8
missing	0.3	0.3	1.8	0.5	17.6	3.5
Social caste, %						
General caste	5.2	10.8	29.6	1.6	12.8	3.3
Other backward	43.2	43.5	9.4	20.3	35.0	16.7
Scheduled caste	33.8	33.9	32.7	6.8	11.0	8.8
Scheduled tribe	17.2	11.0	5.9	17.5	41.0	11.7
DK/ missing	0.7	0.7	22.4	53.8	0.1	59.4
Economic class, %						
BPL	98.3	98.5	57.2	4.6	55.7	85.0
Others	0.5	0.4	28.8	0.1	44.0	10.6
DK/ missing	1.2	1.1	14.0	95.3	0.2	4.4
Area, %						
Rural/tribal	76.9	78.1	80.9	92.7	88.6	Not available
Urban	23.1	21.7	6.3	7.3	11.4	
Missing	0.1	0.2	12.8	0.0	0.0	
Type of transfer, %						
IFT	8.7	9.9	11.3	3.2	2.4	Not available
Non-IFT	91.4	90.1	88.7	96.8	97.6	
Delivery en-route, %						
At pick up site	0.6	0.7	1.5	2.7	1.7	1.7
In ambulance	0.7	0.7	3.3	1.5	0.9	1.8
Type of hospital, %						
Public	72.8	71.4	94.7	88.2	70.1	Not available
Private	13.7	16.0	2.5	3.2	27.4	
Missing	13.5	12.7	2.8	8.6	2.5	
Obstetric emergency, %	3.4	3.3	9.3	4.4	5.6	2.7

*BPL* Below poverty line; *IFT* Inter-facility transfer, *DK* Don’t know

### Obstetric emergencies transported by ‘108’

Table 4 presents further detail on the type of obstetric emergency. The most common obstetric emergency was abnormal presentation of the foetus (Telangana, Andhra Pradesh and Gujarat), bleeding in pregnancy (Himachal Pradesh and Assam), and medical conditions complicating pregnancy in Chhattisgarh. Among pregnant women transported for obstetric emergency, the proportion of inter-facility transfers ranged from 4.5 % in Gujarat to 25.5 % in Himachal Pradesh.

**Table 4:** Characteristics of obstetric emergencies transported by ‘108’ April 2013 to March 2014

	Telangana, *N* = 3,570	Andhra Pradesh, *N* = 4,837	Himachal Pradesh, *N* = 2,316	Chhattisgarh *N* = 2,660	Gujarat *N* = 15,065	Assam *N* = 4,040
Area, (%)						
Rural/tribal	2580 (72.3)	3646 (75.4)	1789 (77.3)	2436 (91.6)	12974 (86.1)	Not available
Urban	985 (27.6)	1177 (24.3)	270 (11.7)	224 (8.4)	2091 (13.9)	-
Missing	5 (0.1)	14 (0.3)	257 (11.1)	0 (0.0)	0 (0.0)	-
Type of transfer, (%)						
IFT	531 (14.9)	784 (16.2)	591 (25.5)	348 (13.1)	681 (4.5)	Not available
Non-IFT	3039 (85.1)	4053 (2.8)	1725 (74.5)	2312 (86.9)	14384 (95.5)	-
Type of complication, %						
Abortion	137 (3.8)	301 (6.2)	292 (12.6)	113 (4.3)	412 (2.7)	288 (7.1)
Abnormal						
Presentation	1156 (32.4)	1520 (31.4)	115 (5.0)	712 (26.8)	8369 (55.6)	788 (19.5)
Bleeding in pregnancy	596 (16.7)	818 (16.9)	1339 (57.8)	133 (5.0)	2458 (16.3)	1782 (44.1)
Eclampsia/ convulsion	255 (7.1)	370 (7.7)	59 (2.6)	65 (2.4)	220 (1.5)	396 (9.8)
Fever	93 (2.6)	136 (2.8)	0 (0.0)	47 (1.8)	136 (0.9)	0 (0.0)
Foetal loss	71 (2.0)	106 (2.2)	41 (1.8)	227 (8.5)	62 (0.4)	27 (0.7)
Medical condition						
complicating pregnancy	733 (20.5)	774 (16.0)	356 (15.4)	1214 (45.6)	1011 (6.7)	36 (0.9)
Previous caesarean	291 (8.2)	481 (9.9)	65 (2.8)	127 (4.8)	1219 (8.1)	416 (10.3)
Precious pregnancy	238 (6.7)	331 (6.8)	49 (2.1)	22 (0.8)	1178 (7.8)	307 (7.6)
Distance call to site,^a^ Kilometres ; Median (IQR)	10 (3–18)	10 (3–18)	9 (1–17)	8 (1–17)	11 (6–17)	Not available
Distance site to hospital,^a^ Kilometers; Median (IQR)	21 (11–32)	21 (12–31)	17 (7–30)	10 (4–20)	15 (9–23)	Not available
Time call to site,^a^ minutes ; Median (IQR)	23 (12–35)	24 (12–37)	32 (15–56)	23 (12–37)	23 (15–33)	Not available
Time site to hospital,^a^ minutes ; Median (IQR)	38 (23–56)	37 (23–55)	60 (35–95)	25 (14–43)	24 (15–36)	Not available
Time call to hospital,^a^ minutes ; Median (IQR)	73 (54–98)	75 (55–97)	91 (63–146)	60 (41–86)	57 (43–75)	Not available

*IFT* Inter-facility transfer, *IQR* Interquartile range

^a^ *N* varies- excludes deliveries by EMT that were not transported or missing values

**Fig. 1:**
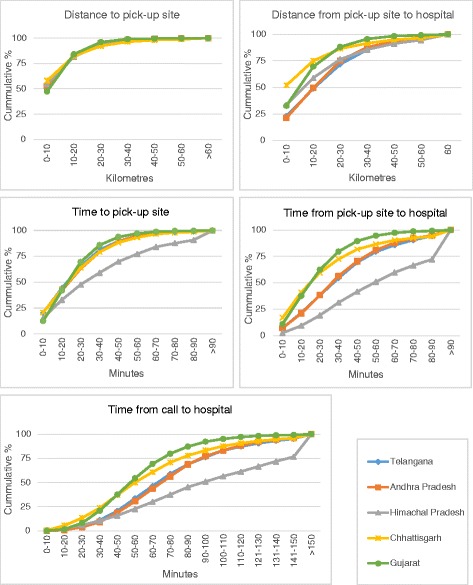
Distance and time travelled by 108 ambulance for women with obstetric emergency

Distances travelled and time taken by ‘108’ ambulances to transfer pregnant women with emergency are shown in Table 4 and Fig. 1. Ambulances travelled less than 10–11 km to reach half the pregnant women with emergency across all the study states. However median distances to the health centre were between 10 km in Chhattisgarh to 21 km in the states of Telangana and Andhra Pradesh. The time taken in travel to reach the pregnant women, and to the hospital, was lowest in Chhattisgarh and Gujarat. Although distances in Himachal Pradesh were not the highest, the travel time was the longest compared to the other states. The median time from call to ‘108’ and reaching a hospital ranged from 60 min to 90 min in all the states and the 75th percentile in Himachal Pradesh was high at 150 min. The median distances and times travelled by pregnant women with emergencies were up to 5–10 kms further and 10–20 min longer than pregnant women without emergency (data not shown).

## Discussion

The ‘108’ scheme was the only large publically financed scheme and the main free transport of pregnant women in these states for the period of study. Some women did not use ‘108’ ambulance despite being provided one. These women would have either delivered or left using other means of transport before ‘108’ ambulance reached the location. The calls that were not assigned ambulance was a very small proportion of all calls to ‘108’. A ‘108’ ambulance is not provided to a pregnant women if the ambulance of that catchment area is on a journey to transfer another client.

The analysis reported here estimated that between 9.0 % and 20.5 % of pregnant women in the population in 2013–2014 were transported by ‘108’. This suggests that ‘108’ was the choice of transport for less than one-tenth to one-fifth of pregnant women. However, pregnant ‘108’ users were more likely to be from below the poverty line and scheduled caste and tribes compared to the general population, signifying that relatively more needy women were utilising ‘108’ when pregnant, compared to the more well off in most states. The DLHS-4 survey in 2012–13 found that the proportion of institutional deliveries was 94.1 % in Telangana, 88.5 % in Andhra Pradesh and 77.8 % in Himachal Pradesh [27–29]. Of the institutional deliveries, it was reported that 7.4 % in Telangana, 12 % in Andhra Pradesh and 20.8 % in Himachal Pradesh used an ambulance for transport to hospital [27–29]. These figures are broadly in line with the findings reported here. Between 4.2 % and 10.7 % of home deliveries stated non-availability of transport as the reason for not delivering in any health institution [27–29].

The number of pregnant women transported by ‘108’ in (undivided) Andhra Pradesh increased from 65,009 in 2007–08 to 323,495 in 2009–10 [30] and continued to be approximately 300,000 till 2013–14. Simultaneously in (undivided) Andhra Pradesh, the proportion of pregnant women transported by ‘108’ who had any complication or emergency reduced from 40 % in 2007–08 [22] to 3.3 %, in 2013–14. This is encouraging for the scheme since, as desired, more pregnant women were accepting ‘108’ ambulance services for normal deliveries than just for obstetric emergencies.

While the transport services may transport all pregnant women, irrespective of the high-risk or actual complication, the success of such a system should be measured by the proportion of all pregnant women with complications who were transported to an appropriate referral level [19]. In our study, the estimated proportion of obstetric emergencies in the population transported by ‘108’ was 12.7 % in Himachal Pradesh, 7.2 % in Gujarat and less than 3.5 % in other states. It is thus unlikely that 108 ambulances had significant impact on maternal death due to complications in pregnancy. A previous study estimated that GVK EMRI ‘108’ ambulance services contributed to a 15 % reduction in the MMR in 2009 in (undivided) Andhra Pradesh by facilitating the proportion of institutional deliveries and probable lives saved [30]. However, the analysis was a crude estimation without accounting for the proportion of obstetric emergencies transported, and other social, economic and health system factors. Another study conducted in the state of Punjab in 2013, observed that there was sudden increase in institutional deliveries immediately after initiation of ‘108’ ambulance service, but the adjusted analysis suggested that ‘108’ did not significantly contribute to this increase in institutional delivery [31].

However most complications arise during or after delivery [32].. While most of the pregnant women were transported by ‘108’ during normal labour pains, it is likely that some may have developed complications after reaching the hospital. In DLHS-4, 23.8 %, 19.9 % and 45.6 % pregnant women in Telangana, Andhra Pradesh and Himachal Pradesh respectively had a complication during delivery, and 15.8 %, 21.7 % and 21.0 % post-delivery respectively [27–29]. It is also possible that women with emergency preferred to use their own vehicle or other faster means of transport than waiting for ‘108’. A study in three districts of (undivided) Andhra Pradesh in 2009 showed that 43.5 % of patients (including pregnant women) admitted to causality departments used ‘108’ ambulances, and 56 % of the ‘108’ users resided within 20 kms of the hospital. Only 10 % of the non-users called ‘108’, but an ambulance was not available for them. Among the non-users of ‘108’, 67 % hired a private taxi/auto, 20 % used their own vehicle, 7 % used other private ambulances and the remaining used other modes of transport [33].

Among obstetric emergencies transported by ‘108’ in this analysis, only 4.5 % in Gujarat and between 13 % and 25 % in other states were inter-facility transfers. It appears that the pregnant women who developed complication while at home either called ‘108’ and left for higher level facility directly, or in case of inter-facility transfer they used other means of transport without waiting for a ‘108’ ambulance. The Chiranjeevi scheme in Gujarat could also have contributed to a low proportion of inter-facility transfers in the state. The Chiranjeevi scheme provides free normal and surgical delivery care to the poor close to their home, with private hospitals providing CEmOC services. This could possibly have reduced the need of referral and transfer in case of an emergency [34].

We observed a higher proportion of pregnant users of ‘108’ in Himachal Pradesh including for inter-facility transfers compared to the other states. Himachal Pradesh has a hilly terrain and the time taken to travel to reach hospital was longer compared to similar distances in other states. The availability of EMT and stabilizing care during the longer time of travel may be the reason for higher usage of ‘108’ ambulances in Himachal Pradesh. However other social factors also need to be explored.

The ‘108’ ambulances took longer than the targeted 20 min to reach a hospital for more than 60 % to 80 % pregnant women in the study states. The women with obstetric emergencies travelled larger distances and in longer time compared to women without emergencies. This was probably due to the fact that the emergency cases had to be transported across longer distances from rural towns to district headquarters. Overall time from call to reaching the hospital was less than 2 h for 61 % pregnant women with emergency in Himachal Pradesh and between 89 % and 98 % in other states.

A main limitation of our study is that details on the type of emergency were based on the clients claim, or the doctor’s report (in case of IFT) or the diagnoses by the EMT. Thus the skill for diagnoses made for emergencies was not uniform and may be inaccurate. This may affect the validity of the reporting. It cannot be estimated if this would have led to over-estimation or under-estimation of the proportion of obstetric emergencies. Details on the type of emergency were not available for clients who were not assigned an ambulance and for most of the clients who did not use an ambulance despite being assigned one. Thus the proportion of obstetric emergencies for these two groups could not be computed. The assumption used for estimating population proportion of obstetric emergencies—that atleast 15 % of all pregnancies are likely to develop complications that may require higher level of care—is debated and may not be accurate for the study areas. Some women would have used ‘108’ service more than once and be counted more times for the same incidence. There was no mechanism to identify these in the database. We anyhow assume that these will be a very small proportion to affect the overall results. Data for treatment given en-route, and doctors’ notes on inter-facility transfers were mostly not recorded in the ‘108’ database. Thus this aspect of the ambulance service could not be studied. Lastly, data obtained from the state of Assam missed some key variables and the information could not be obtained even after repeated requests. Thus only a few variables could be analysed.

Despite limitations in the dataset, this research had several strengths. It involved a unique analysis where the proportion of all women in the population transported by ‘108’ was computed —both for pregnancy and pregnancy with obstetric complication and emergency. We studied their social, economic and geographical distribution which helped in assessing the coverage by ‘108’ at the population level. We also had an advantage that the key national surveys for the country (SRS, DLHS-4, ALHS) for maternal health were conducted during the study period, thus we could triangulate datasets to drive population based interpretations.

India has achieved targeted institutional delivery rates and the debate now revolves around the role of ‘108’ in transporting normal labour cases compared to obstetric emergencies, and strategies to increase the impact on maternal and peri-natal mortality at lower cost. One study has shown that ‘108’ spent $17 on the operational cost for the transport of one case [23]. An important question is whether ‘108’ type ambulances, which are sophisticated vehicles, are required to transport women in normal labour. Studies in Nigeria and India suggest that improving transport to EmOCs does not necessarily require ambulances [19, 35]. Studying the morbidity patterns during transport and after admission, and outcomes of pregnancy among users and non-users of ‘108’, will help assess the effectiveness of transport for normal labour or obstetric emergencies.

Another important question is what should be done when the number of users of ‘108’ plateaus over time, as is evident in Telangana and Andhra Pradesh after 10 years (Source: annual records from GVK-EMRI). Does India require interventions to further increase the use of ‘108’ ambulances for all, and to what extent? Will that be cost-effective? Or does India require to rationalize the use of ‘108’ by offering free services for the poor and some fee for others? Telangana and Andhra Pradesh are piloting an intervention to call the potential users of ‘108’ in the last month of pregnancy to plan the transport and place of delivery beforehand. They foresee that this relationship will increase the use of ‘108’ services and also help in planning ahead for the pregnant women with high-risk or any complication in pregnancy. (Source: Expert from GVK-EMRI). Evaluation of this pilot may provide insight into the acceptance of ‘108’ services, preferences, cost and, potential and necessity of increasing the usage of ‘108’.

There is also the possibility of integrating ‘108’ services with other publically financed transport intervention models in India. Haryana Swasthya Vahan Sewa and ‘102’ ambulances system utilize the existing ambulances at the government health institutions for inter-facility transfers, drop back to homes and other elective pregnancy transports [21, 36]. These can reduce the burden on ‘108’ making them more available for emergency transports requiring stabilizing care. Another Janani Express model in Madhya Pradesh utilizes local private taxi operators to transport pregnant women on subsidised rates although without any supportive medical treatment. These are very successful in remote places. It is suggested that these interventions can be combined with centrally managed ‘108’ ambulance services to increase the reach to the wider population [21].

## Conclusions

The ‘108’ ambulances were used by less than one-fifth of women in 6 Indian states estimated to be pregnant over the period 2013–14. Use was more prevalent among the poor, and lower social and economic sections of the population. Although ‘108’ is assumed to play a pivotal role in providing pre-hospital stabilizing care in obstetric emergencies, only a small proportion of journeys made by pregnant women were for complications or emergency in pregnancy. Although there is a large proportion of pregnant women who do not use ‘108’, it is probable that they prefer other modes of transport. Further community- based studies are required to study the knowledge, and preferences of pregnant women in different sections of society and to assess the potential of increasing or rationalising the use of ‘108’ services.

## Abbreviations

AHS: Annual Health Survey
DLHS: District Level Household Survey
EmOC: Emergency Obstetric care
EMT: Emergency Medicine Technician
GVK-EMRI: GVK - Emergency Medicine Research Institute
IFT: Inter-facility transfers
SRS: Sample Registration System
